# Medical Research Volunteer Program (MRVP): innovative program promoting undergraduate research in the medical field

**DOI:** 10.1186/s12909-016-0670-9

**Published:** 2016-06-06

**Authors:** Michael M. Dagher, Jessica A. Atieh, Marwa K. Soubra, Samia J. Khoury, Hani Tamim, Bilal R. Kaafarani

**Affiliations:** Department of Psychology, American University of Beirut, Beirut, 1107-2020 Lebanon; Department of Biology, American University of Beirut, Beirut, 1107-2020 Lebanon; Department of IT Academic Core Processes & Systems, American University of Beirut, Beirut, 1107-2020 Lebanon; Abu Haidar Neuroscience Institute, American University of Beirut, Beirut, 1107-2020 Lebanon; Department of Internal Medicine, American University of Beirut, Beirut, 1107-2020 Lebanon; Department of Chemistry, American University of Beirut, Beirut, 1107-2020 Lebanon

## Abstract

**Background:**

Most educational institutions lack a structured system that provides undergraduate students with research exposure in the medical field. The objective of this paper is to describe the structure of the Medical Research Volunteer Program (MRVP) which was established at the American University of Beirut, Lebanon, as well as to assess the success of the program.

**Methods:**

The MRVP is a program that targets undergraduate students interested in becoming involved in the medical research field early on in their academic career. It provides students with an active experience and the opportunity to learn from and support physicians, clinical researchers, basic science researchers and other health professionals. Through this program, students are assigned to researchers and become part of a research team where they observe and aid on a volunteer basis. This paper presents the MRVP’s four major pillars: the students, the faculty members, the MRVP committee, and the online portal. Moreover, details of the MRVP process are provided. The success of the program was assessed by carrying out analyses using information gathered from the MRVP participants (both students and faculty). Satisfaction with the program was assessed using a set of questions rated on a Likert scale, ranging from 1 (lowest satisfaction) to 5 (highest satisfaction).

**Results:**

A total of 211 students applied to the program with a total of 164 matches being completed. Since the beginning of the program, three students have each co-authored a publication in peer-reviewed journals with their respective faculty members. The majority of the students rated the program positively. Of the total number of students who completed the program period, 35.1 % rated the effectiveness of the program with a 5, 54.8 % rated 4, and 8.6 % rated 3. A small number of students gave lower ratings of 2 and 1 (1.1 % and 0.4 %, respectively).

**Conclusion:**

The MRVP is a program that provides undergraduate students with the opportunity to learn about research firsthand as they volunteer and aid in different research projects. This program also provides faculty members with the help to conduct their research projects and opportunity to influence future generations. It was shown that so far the MRVP has been successful in reaching its goals, for both students and faculty.

## Background

Research is an integral part of science that develops not only the field itself, but also the researcher. Without it, there could be no progress. As such an important part of the field, it comes as a surprise to find that its value is not better highlighted during students’ undergraduate education. Research is a process that must be mastered to ensure the continuity of any science. It follows that students should be exposed to research as soon as possible, providing them with enough time to become truly proficient [20]. Research exposure at such an early academic level serves more than just to engender an interest in research. Several studies have shown an increase in student interest and involvement in their academic field after exposure to research [7, 10, 11]. It also provides them with a platform from which to begin their professional socialization [6, 14, 15]. Research exposure also serves as a means by which to clarify a specific career path [6, 8, 17, 18]. Students, who engaged in research, reported increased development of their critical thinking [3, 16, 21], specific research skills [3, 5], communication [6, 9, 13, 20], self-confidence in their abilities [3, 7, 17] and sense of self-efficacy [1, 4, 19]. Research also provides the student with the chance to move beyond simple textbook learning and to learn in a more hands-on environment. This experience greatly enriches the education of these individuals.

Exposing undergraduate students to research not only benefits them but also the faculty and researchers who provide this exposure [21]. By working with undergraduate students, faculty members get the chance to refine and shape the scientific minds of the future. In fact, this is often listed as one of the top motivating factors for faculty to invest in students and their education [20]. Another highly prominent benefit to the researchers themselves is the added input and contribution that the students bring to the research project. As expected, the longer a student can contribute to a project, the more meaningful the contribution can become. As such, many faculty members encourage research exposure from as early on as Freshman year [21].

Although all the aforementioned benefits apply to all students, it holds true for undergraduates interested in pursuing a career in the medical sciences more than most. With the dominant wave of evidence-based learning and practice in the medical field, the ability to understand and appreciate research is crucial. Research exposure in the medical field provides students with vital skills such as the ability to understand, interpret and evaluate evidence, research methodology and medical experimental results. These skills allow undergraduates to become active members of the professional medical research community and not just passive spectators.

Most educational institutions lack a structured system that provides students with this highly valuable research exposure in the medical field. This lack constitutes a major shortcoming of the undergraduate education leading up to the medical profession. This paper details a solution to this limitation: the *Medical Research Volunteer Program (MRVP).* This program aims to systematically and reliably provide research experience to undergraduate students interested in entering the field of medicine. The primary objective of this paper is to describe the structure and function of the MRVP and to promote and facilitate its replication in other universities around the world. Moreover, the secondary objective is to assess the success of the program by analyzing data about participation and satisfaction with the program.

## Methods

Following is the description of the methodology followed for each of the two primary and secondary objectives of this paper.

### Primary objective

The MRVP is a program that targets undergraduate students interested in becoming involved in the field of medical research early on in their academic career. It provides these students with an active experience and the opportunity to learn from and support physicians, clinical researchers, basic science researchers and other health professionals. Through this program, students are assigned to researchers and become part of a research team where they observe and aid on a volunteer basis. For many students, the MRVP will provide their first exposure to the field of research. As students help in the various tasks assigned to them, they will witness first-hand the extensive work that goes into scientific research.

The MRVP was established at the American University of Beirut (AUB) in Lebanon. AUB is one of the leading universities in the region, founded in 1866. AUB has a diverse population of students. There are 8712 students enrolled at AUB (September 2015). Almost one quarter of these students are internationals.

### The stages of the MRVP development

The MRVP has passed through three major stages as it developed. Each of these stages is described below.

- ***The Dormant Phase***

The dormant phase of the MRVP was initiated in the Fall of 2013 by one of the authors. It entailed matching students who showed outstanding potential to faculty members who were willing to welcome undergraduate student volunteers into their research teams. Although at the time there was no structured program guiding these matchings, this phase served as a major stepping stone for the program.

b. ***The Pilot Study***

Following the successful experience of the dormant phase, the pilot phase was conducted by the authors of this paper during the summer of 2014. It entailed the development of the program’s guidelines and procedures. It was in this phase that both the MRVP website and the accompanying portal were developed. The pilot study marked the official inauguration of the MRVP.

c. ***The Current Stage***

After the base of the MRVP had been firmly established, the current phase of the program began in the Fall semester of 2014. This program relies on four major pillars: the students, the faculty members, the MRVP committee, and the online portal. The following four sections provide more details about each of the four pillars.

#### Students

The MRVP is open to undergraduate students at AUB who are interested in pursuing a career in the medical field. This is not a program that can cater to the needs of all students. As such, certain inclusion criteria were developed.

One criterion for admission to the MRVP is that the student must be interested in medical research and willing to commit to the requirements of the program. Students from a variety of majors (Biology, Chemistry, Chemical Engineering, Environmental Health, Medical Lab Technology, Nutrition, and Physics) are eligible to apply. In addition, all premedical students, regardless of major, are eligible to join the MRVP. All applicants must have completed a minimum of 24 credits at the sophomore level and have a cumulative average of 78 % or above. Finally, the applicants must provide at least 8 h/week.

Students’ cooperation and adherence to the instructions given to them is vital to the success of the program. Although each research project is governed by its own specific set of rules based on the nature of the research, there are certain guidelines that every student participating in the MRVP should follow. Upon starting work on a research project, students are expected to contribute to the best of their ability in a committed and ethical manner.

#### Faculty

All faculty members in the Faculty of Medicine with professorial rank and active ongoing research projects are eligible to participate in the MRVP. The faculty members take the role of mentors to the students and facilitate the students’ learning process by providing supervision, guidance, and support. As such, participating faculty members must have sufficient time to invest in the program. In addition, members should allocate suitable tasks for each student based on their skills, expertise, interests, and background.

#### Committee

The MRVP is headed by a committee that is responsible for overseeing the program. The committee is composed of a combination of faculty members (from both the undergraduate level and the Faculty of Medicine) and student representatives. The roles of the MRVP committee include, overseeing the process and ensuring the success of the program, recruiting students and faculty members to the program and matching students and faculty members based on interests and needs of both. The committee is also responsible for following up on the students’ progress in their volunteer work, resolving any arising conflicts throughout the process and continuously evaluating and developing the program.

#### Portal

Developed in coordination with the Information Technology (IT) department at AUB, the portal is an online program that aids in the compilation of the needed data and facilitates the management of this data. It provides an efficient way for both faculty and students to provide their information. The portal grants the MRVP committee access to this information and allows it to be easily managed and manipulated during matching.

### The MRVP process

The MRVP process includes the following steps.

1. **Student Orientation**

The MRVP committee holds a 2 h student orientation before the beginning of each MRVP call. Any student may attend this orientation session although the target audience is primarily those students who have successfully fulfilled all the inclusion criteria previously discussed. It serves to inform students of the program’s procedure and their roles and responsibilities. This orientation also serves to dispel any misconceptions about the program and provides students with an open floor where their questions and concerns can be addressed. Students are also provided with important dates and deadlines for the MRVP. The orientation also serves to teach the students how to interact with the online portal. A live demonstration is included to ensure that all the functions of the portal are explored.

2. **Student Application**

The next step of the process is to collect enough information about the student to understand what type of research he/she is looking for and what role they wish to play. The application form gathers basic personal information as well as information about applicants’ academic records, research experiences, research interests, and time availability. The data gathered through the applications are used to construct a regularly updated database of student profiles allowing students to be promptly assigned to the right research projects. Students apply to the program by creating an online profile that contains all the required information. Applications are time restricted so students only have access to this function at specific predetermined times.

3. **Faculty Information**

Faculty members are prompted to fill out an online form by a mass email and announcements on the institution’s website. This form collects information including contact information such as specialty, current research projects as well as the number of students they are willing to work with and any specific background requirements they wish these students to have. Some faculty members may have more than one active project at any given time and some projects can accommodate more than one student volunteer. Similar to students, each faculty member also provides this information via the generation of an online profile on the portal. This information is also compiled into a database.

4. **Matching**

Matching is the process through which students get paired with projects. Using the information available in the compiled database, the portal generates suggestions of potential student-project matches based on different factors, mainly: research interests, number of proposed interests, availability of projects, as well as time of student’s profile creation. Students are prioritized on a first-come-first-served basis. The portal also assigns alternative matches: cases in which students are available but their research interests are different than the ones needed. The system does not automatically match the students to the projects, it only suggests potential and alternative matching. The matching must be made by members of the MRVP committee via the portal. Both the students and researchers are informed of the details of the match, via an automated email notification. Matching is completed in a timely manner, usually within two days after the application deadline. In this way, students can begin their MRVP experience with a minimal wait between applying and getting matched.

5. **Work Initiation**

Students are expected to initiate contact with the faculty member after being notified via email that they have been matched to a project. Faculty members and students must discuss the research project and the roles and responsibilities which will be delegated to them. Upon finalizing the agreement between the two parties, the student is required to fill out the “MRVP Initiation” form on the portal. This form includes information about the study and the terms of the volunteer work such as the number of hours of work per week and the role of the student in the research project.

6. **Follow-Up**

A “Follow-up” form is submitted, via the portal, to the MRVP office every two months. These reports give the students a platform to voice their suggestions, comments and complaints as well as the chance to inform the committee of what responsibilities have been allocated to them. The report serves to document what students have been doing and the challenges they are facing. It is also used to inform the MRVP committee of any modifications to the initial agreement between the student and the faculty member. This form contains both open-ended and Likert-type questions that aim to assess the satisfaction of the participating students.

7. **End of Agreement**

Students are required to submit a “Student End of Volunteering” form when they finish their volunteer work. This form is a more detailed version of the reports that students have been submitting to the MRVP office bimonthly. The “Student End of Volunteering” form evaluates both the student’s personal experience and the MRVP in general. Although faculty members are not required to submit a “Follow up” form every two months, they are asked to submit a “Faculty End of Volunteering” form at the end of each student’s volunteering period. This gives the researcher an opportunity to evaluate both the student’s performance and the MRVP in general.

8. **Evaluation and updating the program**

The data from the “Follow-Up” and “End of Agreement” forms are used to assess the success of the program after each MRVP call. Any issues that are brought to the committee’s attention through the feedback are addressed before the beginning of the next MRVP call. In addition, the committee continuously evaluates the program in an attempt to enhance and refine it.

### Secondary objective

To address the second objective about the outcome of the program, a sub-study was carried out to assess student and faculty participation as well as their evaluation of and satisfaction with the MRVP. Data was collected from the portal for students who joined between the summer of 2014 and the summer of 2015 (all stages of the program excluding the dormant phase). Data from the dormant phase was not included as it was a preparatory stage that was used to develop the program. The data collected fell into two main categories of information: student and faculty. Student information included basic demographics, major, application rates, dropout rates and satisfaction with the program. For the faculty, the information collected was limited to the number of participating researchers and the number of projects each researcher was currently accepting students on. The data was analyzed by generating and comparing means. Trends across calls were also analyzed and interpreted.

#### Data collection and measures

Data was collected from both students and faculty members. For students, this information included total number of student applicants and demographics such as gender, major, date of enrollment in the MRVP, and academic year at the time of initial registration. This data was collected from the student profiles found on the MRVP portal. For faculty members, information including total number of faculty profiles and projects was also extracted from the MRVP portal. To measure satisfaction, the “Follow-Up” and “End of Volunteering” forms were used. The “Follow-Up” form, administered to students, contains six questions measured on a 5-point Likert scale to assess satisfaction with the program in general. Similarly, seven questions of similar format assessed the students’ satisfaction with the project they had been matched to. Two open-ended questions allowed the students to summarize the role they had played in the research team and allowed them to voice any additional comments. A final question asked students to rate their overall satisfaction with the program on a five-point Likert scale. The student version of the “End of Agreement” form is comparable to the “Follow-Up” form. Data was collected from the faculty members using the faculty “End of Volunteering” form. This form includes seven questions assessing the researchers’ satisfaction with the program and nine questions assessing their satisfaction with the student volunteer. All these questions are measured on a five-point Likert scale. Open ended questions allow the researchers to voice any additional comments.

#### Data analysis

The data collected from the portal and various forms were analyzed using IBM’s Statistical Package for the Social Sciences (SPSS) version 22.0 (IBM, Inc, Chicago, IL). The data was summarized by generating numbers and percentages. Trends were assessed across time.

## Results

Table 1 summarizes the characteristics of the volunteers who were involved in the MRVP. A total of 211 students applied to the program, with more than half of the applicants being female (55.5 %). The highest number of applicants was in the Summer 2014 call (*N =* 83), while the lowest was in the Summer 2015 call (*N =* 36). The vast majority of the applicants were from the Faculty of Arts and Sciences (82.9 %), followed by the Faculty of Health Sciences (13.7 %), and the minority were from the Faculty of Engineering and Architecture (0.5 %) and Hariri School of Nursing (0.5 %). Within the Faculty of Arts and Sciences, Biology (78.9 %) and Chemistry (12.0 %) students comprised most of the applicants, while Philosophy (0.6 %) students were the least. Regarding the class breakdown, application rates were the highest for juniors (50.7 %) and lowest for sophomores (19.8 %).

**Table 1:** Characteristics of volunteers who were involved in the MRVP

Student characteristics	Number of Students Applied *N =* 211	Number of Matches *N =* 164	Number of Students Dropped *N =* 60
Call^a^			
Summer 2014	83	75 (45.7 %)	30 (50.0 %)
Fall 2014–2015	43	26 (15.9 %)	9 (15.0 %)
Spring 2014–2015	54	30 (18.3 %)	9 (15.0 %)
Summer 2015	36	33 (20.1 %)	12 (20.0 %)
Gender			
Female	117 (55.5 %)	79 (48.2 %)	28 (46.7 %)
Male	94 (44.5 %)	85 (51.8 %)	32 (53.3 %)
Major			
Faculty of Arts and Sciences	175 (82.9 %)	141 (86.0 %)	50 (83.3 %)
Biology	138 (78.9 %)	117 (83.0 %)	44 (88.0 %)
Chemistry	21 (12.0 %)	13 (9.2 %)	4 (8.0 %)
Mathematics	2 (1.1 %)	0 (0.0 %)	0 (0.0 %)
Philosophy	1 (0.6 %)	1 (0.7 %)	1 (2.0 %)
Physics	3 (1.7 %)	3 (2.1 %)	0 (0.0 %)
Psychology	10 (5.7 %)	7 (5.0 %)	1 (2.0 %)
Faculty of Engineering and Architecture	1 (0.5 %)	1 (0.6 %)	0 (0.0 %)
Chemical Engineering	1 (100 %)	1 (100.0 %)	0 (0.0 %)
Faculty of Agriculture and Food Sciences	5 (2.4 %)	4 (2.4 %)	1 (1.7 %)
Agriculture	1 (20.0 %)	0 (0.0 %)	0 (0.0 %)
Nutrition and Dietetics	4 (80.0 %)	4 (100.0 %)	1 (100.0 %)
Faculty of Health Sciences	29 (13.7 %)	18 (11.0 %)	9 (15.0 %)
Environmental Health	7 (24.1 %)	7 (38.9 %)	4 (44.4 %)
Medical Lab Sciences	22 (75.9 %)	11 (61.1 %)	5 (55.6 %)
Hariri School of Nursing	1 (0.5 %)	0 (0.0 %)	0 (0.0 %)
Nursing	1 (100 %)	0 (0.0 %)	0 (0.0 %)
Class			
Sophomore	42 (19.9 %)	31 (18.9 %)	5 (8.3 %)
Junior	107 (50.7 %)	87 (53.0 %)	31 (51.7 %)
Senior	62 (29.4 %)	46 (28.0 %)	24 (40.0 %)

^a^The data found in this subsection under “Number of Students Applied” include those who have applied to more than one call. The total number of students in this subsection is thus greater than 211, the total number of students who applied to the program

The number of matches within each call since the beginning of the MRVP is also presented in Table 1. A total of 164 matches have been completed. There appears to be no significant difference between the numbers of female (48.2 %) and male (51.8 %) students matched. The highest number of matched students was in the Summer 2014 call (45.7 %), while the lowest was in the Fall 2014–2015 call (15.9 %). Similar to the application rates, the Faculty of Arts and Sciences (86.0 %) has the highest matching rate among the faculties, and Biology (83.0 %) has the highest matching rate among the majors within its faculty. The rates of students matched with respect to class is similar to the rates of students applied.

More than half of the students successfully completed the volunteering period in each call (Table 1). Of the total 60 dropped students, the highest was in the Summer 2014 call (50.0 %), followed by Summer 2015 call (20.0 %), and Fall and Spring 2014–2015 calls (15.0 %). The student dropout rates for males (53.3 %) are higher than those for females (46.7 %). With regard to faculty, the majority of dropped students are in the Faculty of Arts and Sciences (83.3 %), of which most are Biology students (88.0 %). In addition, juniors (51.7 %) experienced the highest rates of dropped students, with the lowest rates belonging to sophomores (8.3 %).

Table 2 provides the numbers of registered faculty members and projects in each call. A total of 102 projects were registered in the MRVP. 48.0 % of projects were registered in the Summer 2014 call, 16.7 % in the Fall 2014–2015 call, 20.6 % in the Spring 2014–2015 call, and 14.7 % in the Summer 2015 call. Regarding the faculty member registration, 49.3 % were registered in the Summer 2014 call, 16.0 % in the Fall 2014–2015 call, 22.7 % in the Spring 2014–2015 call, and 12.0 % in the Summer 2015 call, amounting to a total of 75 faculty member registrations. Since the beginning of the dormant phase of the MRVP, three students have each co-authored a publication in peer-reviewed journals with their respective faculty members.

**Table 2:** Number of projects and faculty members registered in each MRVP call^a^

	Projects	Faculty members
Total	102	78
Summer 2014	49 (48.0 %)	37(47.4 %)
Fall 2014–2015	17 (16.7 %)	12(15.4 %)
Spring 2014–2015	21 (20.6 %)	17(21.8 %)
Summer 2015	15 (14.7 %)	12(15.4 %)

^a^Each registered project can continue over more than one call. The numbers of projects and faculty members displayed under each call may include both newly registered and continuing ones from previous calls

### Response to the “End of Volunteering” form

**Fig. 1:**
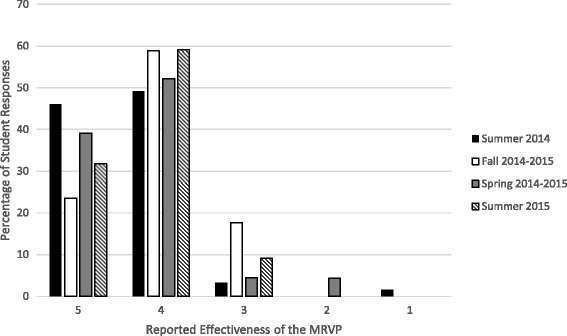
Percentage of the student responses to the question about the effectiveness of the MRVP, with 1 being not effective at all and 5 being extremely effective.*

Figure 1 illustrates the ratings of the effectiveness of the program as reported by the students. The majority of the students provided positive responses and high ratings throughout all the calls. Of the total number of students who completed the program period, 35.1 % rated the effectiveness of the program with a 5, 54.8 % rated 4, and 8.6 % rated 3. A small number of students gave lower ratings of 2 and 1 (1.1 % and 0.4 %, respectively). In the Summer 2014 call, the “End of Volunteering” form was filled out by 63 students, yielding a response rate of 94.0 %. The results showed that 46.0 % of students rated the effectiveness of MRVP with a 5, 49.2 % rated 4, 3.2 % rated 3, and only 1.6 % rated 1. In the Fall 2014–2015 call, none of the students rated the effectiveness of the program with 2 and 1. The response rate was 100.0 %. Twenty-three percent of students rated 5, 58.8 % rated 4, while only 17.6 % rated 3. The overall response rate of the Spring 2014–2015 call was also 100.0 %. More than a third (39.1 %) rated 5, 52.2 % rated 4, and 4.4 % rated 3. Only 4.3 % responded with a 2. Similar positive responses were reported in the Summer 2015 call. Of the total number of students who completed the volunteering period, 66.7 % (*N* = 22) filled out the “End of Volunteering” form, where 31.8 % rated the effectiveness of the program with a 5, 59.1 % rated 4, and 9.1 % rated 3.

**Fig. 2:**
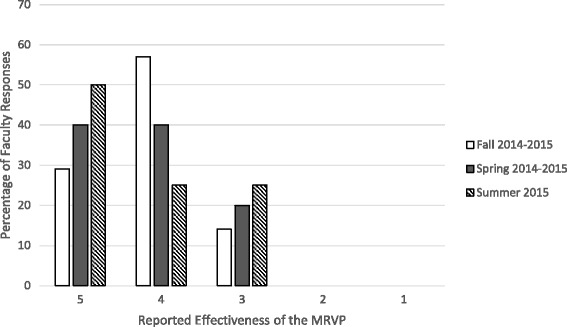
Percentage of the faculty responses to the question about the effectiveness of the MRVP, with 1 being not effective at all and 5 being extremely effective.*

Figure 2 portrays the faculty member response rates concerning the effectiveness of the MRVP. In the Fall 2014–2015 call, the “End of Volunteering” form was filled by seven faculty members, yielding a response rate of 58.3 %. 13.3 % of the respondents rated the effectiveness with a 5, 60.0 % rated 4, and 26.7 % rated 3. The response rate of the subsequent spring semester (58.8 %) was comparable to that of the Fall 2014–2015 call. However, more positive responses were reported, with 40.0 % responding with a 5, 40.0 % responding with a 4, and 20.0 % responding with a 3.

## Discussion

The MRVP is a program that provides to many undergraduate students their first exposure to medical research. Since its launch, the program has directed four calls and matched hundreds of students to projects in need of volunteers. The development of the portal is one of the MRVP’s many milestones that helped the program meet its goals and objectives. The progress and support of the program are dependent on its constant evaluation.

The MRVP was designed to serve the needs of both students and faculty members. Undergraduate students require proper exposure to research and mentorship in the medical field. On the other hand, faculty members require access to a constant supply of assistance to aid them in their research projects. Thus, the MRVP is a program that mutually benefits both parties.

For students, the MRVP provides them with an environment in which they will learn a subject matter in-depth from a hands-on experience and build long-term connections with physicians and researchers. By providing opportunities to participate in every phase of research, the MRVP allows students to learn how to apply scientific methodology and techniques, acquire laboratory skills and improve social skills such as teamwork and oral communication, and strengthen academic credentials. A recent study conducted in 2010 supports the aforementioned based on interviews with students who have engaged in undergraduate research in liberal colleges [12]. Laursen et al. found that the students who participated in research reported gains that can be grouped into six categories: “Personal/professional gains; Gains in thinking and working like a scientist; Gains in becoming a scientist; Gains of skills; Enhanced preparation for career and graduate school; and Clarification, conformation, and refinement of career and educational goals and interests”.

The MRVP is a signature program that provides more than just research opportunities for undergraduates. The program views research as the exploration of the unknown, whether in scientific or in personal terms. Decisions in the scientific community are as important as decisions taken in a career pursuit. Along with academic excellence, a career in the medical field requires life-long commitment and dedication to achieve success. A recent literature review has shown that completing undergraduate research programs may impact the student’s choice of future occupation [2]. Yet, many students follow a career path based largely on academic requirements and minute work experience. The MRVP realizes this lack of experience and works to foster intellectual maturity necessary for career decisions.

Faculty members participating in the MRVP are prestigious physicians whose projects reflect the type of work found in medical research. The MRVP emphasizes the significance of working with physicians at an early stage, allowing undergraduates to capture the reality of the work and dispel any misconceptions about this specific career path. The MRVP also allows students to contact faculty members in our committee for advice on academic and professional pursuits. In addition, a recent review on scholarly activity programs revealed that the students’ work in such programs influences their choice in career paths and interest in research [2]. The program thus highly values the discovery of oneself and works to initiate it at an early stage.

For faculty members, the MRVP provides a constant access to a wide pool of interested students willing to participate and contribute to research projects. It also provides faculty members with the opportunity to impact the minds of future generations by helping talented young students grow professionally and academically. Zydney et al. [21] conducted a study on faculty perception of undergraduate research at the University of Delaware. Results of surveys filled by faculty members showed that 75 % of the respondents perceive their desire to influence young students’ careers to be “important” or “very important” [20]. In addition, an undergraduate student’s fresh point of view can sometimes influence the faculty member’s thinking about the research project. Faculty members have an opportunity to communicate their subject with joy and passion, and, in the long-run, entice new students to their fields.

Finally, the MRVP is a program that aims to improve the overall quality of life at AUB and Lebanon. It contributes to the development of the research culture and promotes an interactive scientific community at AUB by inspiring passion for research in young talented minds. The program also helps researchers to be more productive in their research by providing support. This will ultimately translate into an increase in the productivity and impact of research conducted locally and nationally.

The gender demographics presented in Table 1 show the female to male ratio of students applied to the MRVP to be higher than that of the student population in AUB (1 male:1 female). A significantly higher percentage of male students dropped out of MRVP is also depicted. No previous studies have been found to explain such differences in gender demographics in terms of undergraduate research participation.

A decrease in the number of participating students during the calls following the pilot study is also evident in Table 1. The decrease may reflect the increased difficulty of working on a research project in addition to maintaining high academic performance during the semester. Moreover, after registering for their first call, many volunteers matched with faculty members resume working during the following calls without updating their profiles on the portal, resulting in false negatives. In addition, due to their little experience in conducting research, some volunteers are not clear about what research is. A common misconception held by many students, especially school-leaver entrants, is that medical research always occurs in a lab. The faulty equation of research to lab work has led many students to express dissatisfaction when matched to a project that requires data analysis, proposal writing or manuscript writing. Though dissatisfied, the program evidently served one of its purposes of reflecting to students the possible tedious nature of research projects.

The aforementioned reasons may also explain the student drop out incidence in every call. Table 1 shows no significant difference in the number of students dropped among the calls, as the number dropped is proportional to the number matched in every call. In any case, the high number of students dropping out or disappearing from the program has proved to be a pressing challenge. It generates a decreased tendency of faculty members to participate in the MRVP and ask for more student volunteers in the future, as depicted by the decrease in number of faculty members and projects in Table 2.

The results in Table 1 also show the Biology and Chemistry undergraduates comprising the highest percentages of students applied and matched. These high proportions are comparable to those of Biology and Chemistry pre-medical students at AUB. In addition, junior students constitute the highest number of profiles registered on the portal (Table 1). This may suggest that most students who are interested in the program are those who have found stability in their major and wish to gain better perspective on their academic or professional pursuit.

Like any novel program, it is crucial to objectively assess the success of the MRVP. Since the program is still in its early stages, measuring success in terms of publications will be unreliable. In addition, using the number of participating students and faculty members as a measure has shown to be defective due to false negatives. Our outcome measure is thus the feedback provided by both participating students and faculty. As illustrated by Figs. 1 and 2, the majority of responses provided by students and faculty are positive, which reflect the success and utility of the program.

The MRVP has faced several challenges, mainly developing, maintaining and upgrading the portal, as well as sustaining students and faculty commitment. At the early stage of the portal development, difficulties arose in managing the students’ profiles and projects opened by faculty members. Challenges that affected the follow-up process of the program included the inability to manually match students and manage the status of faculty members’ projects, the inability to insert comments to student profiles, and the difficulty in culminating results to establish a report. Nonetheless, functionalities and features were added to incrementally adapt to the program maturity and to users’ requirements. Committee members now have access to override the portal and manually match projects and manage the status of the projects. In addition, a reporting tab was added to increase the feasibility of reviewing the results of each call and developing reports.

As for sustaining the students and faculty commitment, it has been a challenge throughout all calls. To address the students’ misconception of what research work entails, a student orientation lecture is held as of the 2015 Fall semester. During the first orientation session, which was organized at the beginning of the 2015 Fall semester, an overview of the program and the application procedure was presented. The wide range of research processes were explained to present a clear idea to the interested volunteers what to expect when entering the field of research. In addition, as of the 2015 Fall semester, the Fall and Spring calls have been merged to increase student commitment to the assigned project and program and thus encourage faculty members to recruit students. The extended work period will also provide students with a better chance to publish their findings.

Other challenges included the low response rate of filling out the “Follow Up” forms by the students, which affected the committee’s ability to monitor the progress of each student after the matching process. Moreover, faculty members have reported accountability issues where students are underperforming in the program due to other priorities such as academic responsibilities. To overcome these challenges, the importance of this step is highlighted during the orientation session. In addition, a system of closer follow up with the students is being implemented. Finally, another challenge is the lack of sufficient projects and faculty members registered in the program, which results in a number of unmatched applicants in almost every call. This challenge is expected to be resolved by merging the Fall and Spring semesters in one call, as previously discussed.

To further foster student commitment and promote educational innovation, the “Ibrahim and Loulu Durr Endowed MRVP Award” was established in May 2015. The $1000 award will be granted annually as of 2016 to an AUB Biology or Chemistry student enrolled in the MRVP, as per the wish of the donors. Based on the MRVP committee evaluation, the student awardee must “demonstrate outstanding medical research capabilities particularly in the biochemistry field”.

## Conclusions

The MRVP is a program that provides undergraduate students with the opportunity to learn about research firsthand as they volunteer and aid in different research projects. This program also provides faculty members with the help to conduct their research projects and opportunity to influence future generations. The progress of the MRVP has so far proved to be dynamic, and we anticipate more changes to improve the program’s features and functionalities and to overcome the challenges faced. Discovering one’s career direction at an early stage is one of the program’s main benefits for students, in addition to acquiring and honing research skills which are crucial to both researchers and clinicians. Although the program currently involves only undergraduate AUB students, we envision to expand and collaborate with other universities in Lebanon and the Middle East to promote diversity in the community and expose students to a greater variety of projects. More studies are needed to support our findings and test our undergraduate research program model.

### Ethics approval and consent to participate

The work described in this manuscript does not need ethical approval as indicated by the American University of Beirut Institutional Review Board (IRB). The IRB indicated that this work describes a “program improvement rather than a research protocol”.

### Participants

The participants in the MRVP were undergraduate students who were informed about the program (through orientation session and announcements via email and on social media). The participants willingly participated in the MRVP and filled up all the required forms.

### Consent for publication

Not applicable.

### Availability of data and materials

Data are accessible by contacting the corresponding authors.

## Abbreviations

MRVP: Medical Research Volunteer Program
SPSS: Statistical Package for the Social Sciences

## References

[1] Adedokun OA, Bessenbacher AB, Parker LC, Kirkham LL, Burgess WD (2013). Research skills and STEM undergraduate research students’ aspirations for research careers: Mediating effects of research self-efficacy. J Res Sci Teach.

[2] Bierer B, Chen HC (2010). How to measure success: the impact of scholarly concentrations on students – a literature review. Acad Med.

[3] Chaplin SB, Manske JM, Cruise JL (1998). Introducing freshmen to investigative research—A course for biology majors at Minnesota’s University of St. Thomas. J Coll Sci Teach.

[4] Chemers MM, Zurbriggen EL, Syed M, Goza BK, Bearman S (2011). The role of efficacy and identity in science career commitment among underrepresented minority students. J Soc Issues.

[5] Dunn JG, Phillips DN (1998). Introducing second-year chemistry students to research work through mini-projects. J Chem Educ.

[6] Gates AQ, Teller PJ, Bernat A, Delgado N (1998). Meeting the challenge of expanding participation in the undergraduate research experience.

[7] Halstead J (1997). Council on Undergraduate Research: A resource (and a community) for science educators. J Chem Educ.

[8] Humphreys SM (1997). Summer undergraduate program in engineering research at Berkeley. Paper presented at the Proceedings of the Frontiers in Education Conference.

[9] Kardash CM (2000). Evaluation of an undergraduate research experience: Perceptions of undergraduate interns and their faculty mentors. J Educ Psychol.

[10] Kitto KL (1998). Innovative research and laboratory experiences for undergraduate students.

[11] Krochalk P, Hope E (1995). A framework for integrating faculty discipline-related research with classroom teaching and learning. J Excell Coll Teach.

[12] Laursen S, Hunter A-B, Seymour E, Thiry H, Melton G (2010). Undergraduate research in the sciences: Engaging students in real science.

[13] Mabrouk PA, Peters K (2000). Student perspectives on undergraduate research (UR) experiences in chemistry and biology. CUR Quarterly.

[14] McCurdy DL, Buckner B, Baughman RG (1998). Characteristics of the culture of undergraduate research in a liberal arts and sciences university. Council on Undergraduate Research Quarterly.

[15] Nagda BA, Gregerman SR, Jonides J, von Hippel W, Lerner JS (1998). Undergraduate student–faculty research partnerships affect student retention. Rev High Educ.

[16] Nikolova Eddins SG, Williams DF (1997). Research-based learning for undergraduates: A model for merger of research and undergraduate education. J Excell Coll Teach.

[17] O’Clock PM, Rooney CJ (1996). Exposing undergraduates to research through a mentoring program. J Account Educ.

[18] Sabatini DA (1997). Teaching and research synergism: The undergraduate research experience. J Prof Issues Eng Educ Pract.

[19] Seymour E, Hunter A, Laursen SL, Deantoni T (2004). Establishing the benefits of research experiences for undergraduates in the sciences: First findings from a three-year study. Sci Educ.

[20] Zydney AL, Bennett JS, Shahid A, Bauer K (2002). Faculty perspectives regarding the undergraduate research experience in science and engineering. J Eng Educ.

[21] Zydney AL, Bennett JS, Shahid A, Bauer KW (2002). Impact of undergraduate research experience in engineering. J Eng Educ.

